# A pilot randomised controlled trial of cognitive behavioural therapy for antenatal depression

**DOI:** 10.1186/1471-244X-13-33

**Published:** 2013-01-22

**Authors:** Alison Burns, Heather O’Mahen, Helen Baxter, Kristina Bennert, Nicola Wiles, Paul Ramchandani, Katrina Turner, Debbie Sharp, Joanna Thorn, Sian Noble, Jonathan Evans

**Affiliations:** 1 Centre for Mental Health, Addiction and Suicide Research, School of Social and Community Medicine, University of Bristol, Oakfield House, Oakfield Grove, Clifton, Bristol BS8 2BN, UK; 2 Department of Mood Disorder, University of Exeter, Exeter EX4 4QG, UK; 3 Academic Unit of Child and Adolescent Psychiatry, Imperial College, London W2 1PG, UK; 4 Centre for Academic Unit of Primary Care, School of Social and Community Medicine, University of Bristol, Canynge Hall, 39 Whatley Road, Bristol BS8 2PS, UK; 5 School of Social and Community Medicine, University of Bristol, Canynge Hall, 39 Whatley Road, Bristol BS8 2PS, UK

**Keywords:** Randomised controlled trial, Antenatal depression, Cognitive behavioural therapy, Pregnancy

## Abstract

**Background:**

Few trials have evaluated the effectiveness of psychological treatment in improving depression by the end of pregnancy. This is the first pilot randomised controlled trial (RCT) of individual cognitive behavioural therapy (CBT) looking at treating depression by the end of pregnancy. Our aim was to assess the feasibility of delivering a CBT intervention modified for antenatal depression during pregnancy.

**Methods:**

Women in North Bristol, UK between 8–18 weeks pregnant were recruited through routine contact with midwives and randomised to receive up to 12 sessions of individual CBT in addition to usual care or to continue with usual care only. Women were eligible for randomisation if they screened positive on a 3-question depression screen used routinely by midwives and met ICD-10 criteria for depression assessed using the clinical interview schedule – revised version (CIS-R). Two CBT therapists delivered the intervention. Follow-up was at 15 and 33 weeks post-randomisation when assessments of mental health were made using measures which included the CIS-R.

**Results:**

Of the 50 women assessed for the trial, 36 met ICD-10 depression criteria and were randomised: 18 to the intervention and 18 to usual care. Thirteen of the 18 (72%) women who were allocated to receive the intervention completed 9 or more sessions of CBT before the end of pregnancy. Follow-up rates at 15 and 33 weeks post-randomisation were higher in the group who received the intervention (89% vs. 72% at 15 weeks and 89% vs. 61% at 33 weeks post-randomisation). At 15 weeks post-randomisation (the end of pregnancy), there were more women in the intervention group (11/16; 68.7%) who recovered (i.e. no longer met ICD-10 criteria for depression), than those receiving only usual care (5/13; 38.5%).

**Conclusions:**

This pilot trial shows the feasibility of conducting a large RCT to assess the effectiveness of CBT for treating antenatal depression before the end of pregnancy. The intervention could be delivered during the antenatal period and there was some evidence to suggest that it could be effective.

**Trial registration:**

ISRCTN44902048

## Background

Depression is common in women of childbearing age. One large systematic review found a period prevalence for major depression of 12.7% across the 9 months of pregnancy and 7.1% in the first three months postnatally [1].

There has been considerably more research on postnatal depression and its consequences than on antenatal depression but the latter can also have adverse effects on both mother and baby. Women who experience perinatal depression are more likely to have a poor couple relationship, to self-harm [2-4], and it can affect the developing child independently of the occurrence of postnatal depression [5-8]. Antenatal depression has an impact on neonatal development through several mechanisms. Women who are depressed during pregnancy are more likely to smoke cigarettes, use alcohol and other illicit substances [9,10], to experience preeclampsia and other obstetric complications [11,12]. The consequences of these to the developing foetus may include low birth weight, preterm delivery, and reduced motor activity [13-17].

Many of those depressed in pregnancy do not recover and depression continues postnatally. One study found that 50% of women with high depression scores at 2 months postnatally had high depression scores at 32 weeks antenatally [6]. Some of the consequences attributed to postnatal depression could therefore be due to antenatal depression. Identification and treatment of depression at this time has therefore become a health service priority and has been recommended in National treatment guidelines in the UK (NICE ) [18].

There have been a number of studies which have shown psychological and psychosocial treatments to be effective in improving mood postnatally [19-21]. In contrast, there have been few studies on the treatment of antenatal depression that aim to improve depression before the end of pregnancy and only one small trial using interpersonal therapy [22]. Although there are a number of other studies that begin during pregnancy [23,24] their primary aim is to prevent postnatal depression and other adverse postnatal outcomes rather than to successfully treat depression before the end of pregnancy.

The antenatal period provides a unique opportunity to identify and treat depression, as there is contact with general practitioners (GPs) and midwives from the first trimester, and there may be fewer practical barriers to treatment at this time, compared to postnatal depression when the mother has a young infant to care for. If the consequences for child development are to be prevented, then treatment needs to be prompt in order to improve mood before the end of pregnancy.

The choice of intervention in pregnancy is complicated by the need to consider the foetus as well as the mother. Guidance in the UK for treating antenatal depression recommends cognitive behavioural therapy (CBT) or interpersonal therapy (IPT) for severe or moderate depression in those with a previous history. Although the efficacy of CBT in the treatment of depression has been established outside the antenatal period, the effectiveness and feasibility of this approach and implementing it within the healthcare system during pregnancy is unknown. There are several reasons why this extrapolation to pregnancy, of treatments that are effective at other times, is inadequate. First, the risk benefit ratio for antidepressant treatment differs at this time [25,26]. There are known adverse consequences for the developing foetus of some antidepressants [27,28], an association with a lower gestational age at birth and an increased risk of preterm birth [29] and the acceptability of pharmacological treatment during pregnancy is lower [26]. Therefore the consequence of this is that fewer women are using antidepressants during pregnancy than any other time therefore CBT is more likely to be delivered in the absence of antidepressants whilst the efficacy of this is known the effectiveness in this context is unknown. Second, the context in which the treatment is offered is different. There is an assumption that standard CBT approaches will work but some adaptations in content and delivery are needed. There are unique demands to a pregnant woman’s time, attention and energy, which may have an impact on the delivery of CBT. There are particular concerns during this period such as pregnancy specific worries and rumination is heightened along with interpersonal and social support needs [30]. Finally, there is a more urgent need to provide a timely intervention as a delay, could compromise any benefits there may be on foetal development.

With this in mind we conducted a pilot RCT to assess the feasibility for a large-scale RCT of individual CBT in the treatment of depression by the end of pregnancy by piloting procedures for recruitment, assessment and randomisation to treatment, and the delivery of up to 12 sessions of CBT before the end of pregnancy. Data collection procedures were also piloted and included an assessment of the most appropriate way of collecting health care resource use for this population.

## Method

### Setting

All Midwives in North Bristol, UK, a mainly urban setting with some areas of high deprivation, were approached and invited to refer women to the trial. Ethical approval for the trial was given by Southmead National Health Service Research Ethics Committee (09/H0102/75).

### Inclusion/exclusion criteria

Eligible participants were women over 16 years of age who were between 8 and 18 weeks pregnant and who screened positive on a 3-question depression screen [31]. This is routinely used by the midwives as recommended in National guidance, where a positive depression screen is answering yes to either or both of the symptoms questions and yes to wanting help. Women were excluded if they were currently receiving CBT or any individual or group psychological therapy for depression or if they had a psychotic illness. Women who did not have sufficient command of English to complete the questionnaires or benefit from an individual talking therapy without an interpreter were also excluded due to the small scale of the pilot trial.

### Recruitment

Women were recruited at their midwife ‘booking appointment’. These appointments are arranged early in pregnancy and in North Bristol 85% of women have had their booking appointment by 14 weeks of pregnancy. We wanted to establish early contact in order to avoid a delay between ‘booking’ and starting CBT, so that the women were able to complete therapy during pregnancy.

At their booking appointment, women were asked to complete a form giving permission to be contacted by the research team. For those consenting to be contacted, and if the midwife indicated on the 3-question screen that the women may be suffering from depression and would like help, she was given a more detailed information sheet about the trial and a leaflet on CBT. The completed permission to contact forms were returned to the research team by post or fax, the women were contacted, the study formally introduced, eligibility checks conducted and an appointment arranged for a baseline assessment. At this appointment, at the woman’s home, the study was explained in more detail, the computerised version of the Clinical Interview Schedule – revised (CIS-R) [32] was completed to confirm eligibility. Those who met ICD-10 criteria on the CIS-R for depression (mild, moderate or severe) were asked to give written informed consent to be randomised.

### Randomisation

Eligible women were randomised to one of two groups: (1) CBT in addition to usual care; or (2) to continue with usual care only. Allocation was concealed through the use of a central randomisation service that was accessed via the internet and used a computer generated code. Minimisation was used to ensure balance between the trial groups on 4 key design variables: age (< or ≥ 18) depression severity (mild, moderate or severe), current symptom duration (< or ≥ 3 months) and history of depression (yes or no). Women were informed of the outcome of randomisation at the end of the assessment by the researcher. The woman’s GP and midwife were informed that they had depression, had agreed to take part in the study and the group to which they were randomised.

### Intervention

The intervention consisted of up to 12 individual sessions of CBT at the woman’s home unless a preference was expressed to be seen elsewhere. Two therapists, one with master’s level experience and the other with doctoral experience in CBT, were trained to deliver the intervention until judged to be competent. Training the therapist to competence consisted of following an intervention manual modified in previous research to fit with the content needs of perinatal depression [33], reviewing and training in key perinatal adaptations, and role plays with the trial Clinical Supervisor (HO), a Clinical Psychologist with specialist perinatal expertise. Modification of the CBT included paying attention to the role of maternal beliefs, the unique environmental constraints surrounding behavioural activation (e.g., small chunks of available time, balancing multiple valued goals and time-sensitive pressures), and incorporating principles and strategies that address the ways to improve communication and social support. Therapist competence was assessed with the Revised Cognitive Therapy Scale [CTS-R) [34]. In addition, 10% of cases were randomly selected each week to be audiotaped for monitoring adherence to treatment by the clinical supervisor. Issues of non-adherence to the treatment model (e.g. being directive, incorporating strategies from other treatments) were addressed in weekly clinical supervision. Therapists were advised on how to refocus treatment within the trial intervention framework.

An adequate ‘dose’ of CBT for the trial was defined beforehand as at least 9 sessions. This definition was based on the findings of a meta-analysis of CBT in preventing depression that found the strongest effect size in clinical trials offering more than 8 sessions [35]. Although there is some evidence that 12 sessions is the minimum length for which there is evidence of clinical efficacy of CBT in moderate and severe depression [36,37], this must be considered alongside the practical constraints involved in conducting therapy in the antenatal period [38].

### Usual care

After booking, women in both trial groups continued to receive usual care from their midwife and GP. For a first time mother this usually included a further 9 appointments with midwives after the booking plus scans (a dating and anomaly scan) or 6 further appointments and scans if they have had previously had a baby. Midwives routinely decide how frequently to meet pregnant women depending on their perceived needs and available resources. Information on this care was collected from medical and maternity records.

### Data collection and outcome measures

Women were assessed at baseline, at 15 weeks and 33 weeks post-randomisation. The primary outcome for the full scale trial, that was piloted here, was recovery i.e. not depressed according to ICD-10 at 15 weeks post-randomisation. The CIS-R is a self-administered computerised interview and gives an ICD-10 diagnosis of mild, moderate or severe depression as well as a total symptom score based on the duration and severity of depression and neurotic symptoms. A total symptom score above a threshold of 12 indicates the individual has a diagnosable mental health disorder, including anxiety [32,39]. The reliability and validity of the CIS-R are comparable with the composite interview national diagnostic interview [40]. The CIS-R was slightly adapted so that participants answering questions relating to symptoms of pregnancy, such as sleep, fatigue and appetite, were asked to distinguish whether their experiences were due to pregnancy or their mood.

Secondary outcomes (collected at baseline, 15 and 33 weeks post-randomisation) for a full scale trial, piloted here, included the Patient Health Questionnaire (PHQ-9, not modified for pregnancy) and Edinburgh Postnatal Depression Scale (EPDS) [41,42] which were both computer administered. Both these measures are commonly used self-report depression measures suggested by NICE guidance to be used as part of the assessment following the 3-question screen. The Short form questionnaire-12 items (SF-12), was used as a measure of quality of life [43], the EQ-5D as a measure of health outcome [44] and the Prenatal Attachment Inventory was used to assess the level, quality and intensity of the bond between a women and her foetus (scores range from 21 to 84 with higher scores indicating increased attachment quality/intensity) [45]. The Metacognitive Awareness Questionnaire was used to measure how aware participants were of the differences in their thinking according to their mood, where higher scores reflect greater meta-cognitive awareness [46]. The number of midwife appointments and GP visits were recorded via patient-reported resource-use questionnaires (RUQs) developed for this study. Midwife appointments and GP visits were identified from medical records, and additional midwife data from maternity notes.

### Statistical analyses

The characteristics of those women who were randomised were described using appropriate descriptive statistics. The proportion of women; recruited to the trial, completing therapy and followed up were calculated, as well as the proportion recovering from depression in each group. Logistic regression was used to compare the difference in the proportion who ‘recovered’ between the two groups as randomised (‘intention-to-treat’ basis), adjusting for the four minimisation variables: age (< or ≥ 18); depression severity (mild, moderate or severe); current symptom duration (< or ≥ 3 months); and history of depression (yes or no). A sensitivity analysis was conducted to examine the impact of missing data on findings using a best /worst case scenario (i.e. assuming that all those who were missing ‘recovered’ or ‘did not recover’). Differences between groups on the continuous CIS-R score were compared using linear regression. Similar regression models were used for secondary outcomes. Odds ratios (OR), or differences in means, 95% confidence intervals (95% CI) and p values are reported.

For the health economic analysis, mean monthly contacts were derived for the number of midwife and GP appointments recorded by the different methods; Wilcoxon signed-ranks tests were used to compare the methods. All statistical analysis was performed using STATA version 12.1.

## Results

### Recruitment

**Figure 1:**
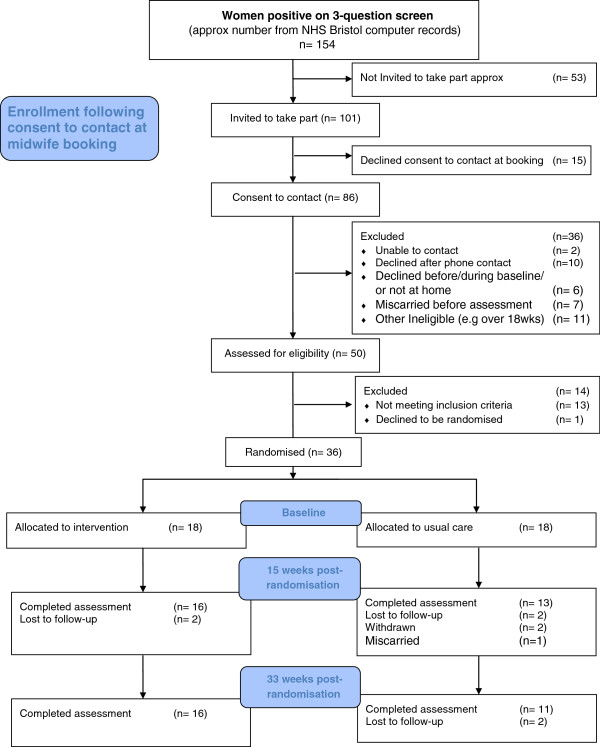
Flow of participant into and through the trial.

According to computerised records from NHS Bristol, during the recruitment period of the study between May 2010 and February 2011, there were approximately 5,409 women who attended booking clinics, of those 154 women screened positive on the 3-question screen for depression and were therefore potentially eligible for assessment for the trial. Figure 1 presents a CONSORT diagram of the flow of women through the trial. The data from NHS Bristol was information on bookings between August 2010 and July 20011 so the figures above are approximations.

In total we received replies from 101 women who gave permission to be contacted (65.6%), of those 15 (14.8%) refused to take part at their booking appointment (main reasons were; too busy and not wanting to take part in research). Of the remaining 86 (85.1%) women who returned a permission to contact form and were potentially eligible, a further 36 (42%) were excluded (main reasons were; declined after researcher contact, over 18 weeks gestation, and miscarriage). A total of 50 women were assessed for the trial, 13 women did not meet the entry criteria as they did not have an ICD-10 diagnosis of depression, one woman did not want therapy (and declined randomisation), leaving 36 women who were randomised into the trial. The average time between booking appointments and randomisation was 21 days (range= 4–59 days, SD= 13).

The midwives invited only 65.6% (101/154) of women who were potentially eligible for assessment for the trial. Reasons given by midwives as to why they failed to refer were ‘misperceptions about the remit of CBT’, ‘woman too unwell for CBT’, some midwives thought ‘CBT suited to those with phobias rather than depression’, some preferred to offer extra appointments with themselves, or some midwives assuming on the basis of the rest of the booking appointment that things were going well for the woman and they did not need to be asked the 3-question screen directly.

### Therapy attendance

Eighteen women were randomised to receive the intervention and 18 to receive usual care. Thirteen of the 18 women receiving therapy completed 9 or more sessions (defined pre-trial as an adequate ‘dose’), giving a completion rate of 72% (CI 49–87.5). The majority of those women (n= 10; 76.9%) attended all 12 sessions. Of the 13 women who continued with therapy, only one woman did not formally complete therapy before giving birth, however she had received 9 sessions whilst still pregnant. The mean gestation when therapy was completed was 32 weeks (range= 22–40 weeks, SD= 4.5).

A number of sessions were cancelled, with many women cancelling with less than 24 hours notice, and the therapist reported a few appointments when the women were not at home (n=10/179 sessions; 5.6%). Four women (22.2%) withdrew from therapy before completion. There were various reasons for their withdrawal, e.g. unable to fit in around work, too busy, disliked CBT, or out of the country.

### Baseline characteristics

The two groups were similar although, not unsurprisingly given the small numbers, some imbalances were apparent in ethnicity, marital status, home ownership, and ever having used antidepressants (Table 1).

**Table 1:** Baseline comparability of the randomised groups

**BASELINE VALUES**	**Intervention**		**Usual care**	
	**n**		**n**	
Age: mean (SD, range)	18	28.2 yrs (5.0: r= 20–36)	18	30.1 yrs (6.2: r= 21–41)
No. of weeks pregnant at assessment: mean (SD, range)		13 .1 wks (3.2: r= 9–18)		12.7 wks (2.2: r= 10–17)
Ethnicity White	13	72.2%	17	94.4%
No Religion	11	61.1%	12	66.7%
Living with Partner	12	66.7%	10	55.6%
Married/ living as married	13	72.2%	10	55.6%
**Socio-economic indicators**				
Housing tenure (owner)	2	11.1%	8	44.4%
Working (full or part time)	12	66.7%	9	50.0%
Educational Qualification (‘ O’ level or equivalent and above)	15	83.3%	16	88.9%
Financial situation: Just getting by/ difficult	12	66.7%	14	77.8%
Car ownership (none)	10	55.5%	12	66.7%
No. of life event in past 6 months: median [IQR)	18	1.5 [0, 3]	17	1 [0, 4]
**Depression History**				
Reported depression in the past	15	83.3%	16	88.9%
Current duration of depression (more than 3 months)	13	72.2%	13	72.2%
Ever used antidepressants before	10	55.6%	15	83.3%
Currently on antidepressants	2	20.0%	5	33.3%
**Clinical variables**				
ICD-10 Diagnosis - Mild	3	16.7%	3	16.7%
Moderate	10	55.5%	10	55.5%
Severe	5	27.8%	5	27.8%
CIS-R Score: median (SD)	18	26.5 (7.9)	18	30.5 (7.5)
EPDS: median (SD)	18	16.5 (4.3)	18	20 (5.1)
PHQ-9: median (SD)	18	16.0 (4.6)	18	16.0 (5.0)
SF-12 Physical component: mean (SD)	18	43.7 (6.6)	17	45.3 (6.8)
SF-12 Mental component: mean (SD)	18	39.9 (7.6)	17	37.5 (8.2)
EQ-5D: mean (SD)	18	0.6 (0.3)	18	0.6 (0.2)
Metacognitive Awareness Questionnaire: mean (SD)	17	33.3 (7.1)	18	36.2 (6.6)

### Follow-up

Overall, 81% (29/36) of women were followed-up at 15 weeks post-randomisation and 75% (27/36) were followed up at 33 weeks post-randomisation (See Figure 1). There was some evidence of differential attrition, such as those in the intervention group were more likely to be followed-up. Follow-up rates were higher in the intervention group at both 15 weeks and 33 weeks post-randomisation (See Figure 1).

### Outcome at 15 weeks – proposed primary

The proportion of women fulfilling ICD-10 criteria for depression in each group, at 15 weeks post-randomisation, is reported in Table 2. The majority, 68.7% in the intervention group no longer met ICD-10 criteria for depression on the CIS-R (‘recovered’) compared to 38.5% of those receiving usual care. Women who received the intervention had a 3.6 fold increased odds of ‘recovery’ at 15 weeks post-randomised compared with those who continued with usual care (Table 3), although, not unexpectedly given the small sample size, the 95% CI surrounding this estimate was wide and included the null.

**Table 2:** Primary Diagnosis on the CIS-R at baseline and follow-up assessments

	**Baseline**		**15 weeks post-randomisation**		**33 weeks post-randomisation (in the postnatal period)**	
	**Intervention (n= 18)**	**UC (n= 18)**	**Intervention (n= 16)**	**UC (n= 13)**	**Intervention (n= 16)**	**UC (n= 11)**
No depression	-	-	11 (68.7%)	5 (38.4%)	13 (81.2%)	4 (36.4%)
Mild depressive episode	**3 (16.7%)**	**3 (16.7%)**	**1 (6.3%)**	**1 (7.7%)**	**1 (6.3%)**	**-**
Moderate depressive episode	**10 (55.5%)**	**10 (55.5%)**	**4 (25%)**	**5 (38.4%)**	**2 (12.5%)**	**5 (45.4%)**
Severe depressive episode	**5 (27.8%)**	**5 (27.8%)**	**-**	**2 (15.3%)**	**-**	**2 (18.2%)**

**Table 3:** Percentage and odds ratio of recovery at 15 weeks post-randomisation

	**N**	**%**	**Odds ratio (**^**1**^**) (95% CI) p-value**	**Odds ratio (**^**2**^**) (95% CI) p-value**	**Odds ratio (**^**3**^**) (95% CI) p-value**
Intervention	11	68.7	3.6 (0.5 to 23.2) 0.2	1.5 (0.3 to 8.0) 0.6	3.9 (0.8 to 17.8) 0.1
Usual care	5	38.4			
Total N	29		29	36	36

(^1^) adjusted for CIS-R score at baseline, age (< or ≥ 18) depression severity (mild, moderate or severe), current symptom duration (< or ≥ 3 months) and history of depression (yes or no).

(^2^) adujsting for CIS-R score at baseline, allocation group, age (< or ≥ 18) depression severity (mild, moderate or severe), current symptom duration (< or ≥ 3 months) and history of depression (yes or no) – assuming all missing ‘recovered’.

(^3^) adujsting for CIS-R score at baseline, allocation group, age (< or ≥ 18) depression severity (mild, moderate or severe), current symptom duration (< or ≥ 3 months) and history of depression (yes or no) - assuming all missing ‘did not recover’.

Sensitivity analyses to examine the impact of missing data were conducted (Table 3). The odds ratio for recovery varied depending on the assumption made with respect to missing data, but the results were consistent with those of the primary analysis, when confidence intervals were compared.

### Continuous CIS-R score at 15 weeks – proposed secondary

As seen in Table 4, continuous CIS-R mean scores were lower for the intervention group compared with the usual care group at 15 weeks post-randomisation. After adjustment for baseline CIS-R score and the 4 key design variables (age, depression severity, current symptom duration and history of depression), the intervention group had a mean CIS-R score that was 10 points lower than those in the usual care group at 15 weeks (Table 4).

**Table 4:** Secondary outcome measures: comparing means between the intervention and usual care groups at 15 and 33 weeks post-randomisation

	**15 weeks post-randomisation**					**33 weeks post-randomisation (in the postnatal period)**				
	**Intervention**		**UC**		**Adjusted difference in means(**^**1**^**) (95% CI) p value**	**Intervention**		**UC**		**Adjusted difference in means (**^**1**^**) (95% CI) p value**
	**n**	**Mean (sd)**	**n**	**Mean (sd)**		**n**	**Mean (sd)**	**n**	**Mean (sd)**	
CIS-R Scores	16	12.4 (9.2)	13	22.3 (11.1)	−10.0 (−16.9 to −2.9) p= 0.007	16	9.8 (8.8)	11	22.4 (11.9)	−12.6 (−21.1 to −4.2) p= 0.005
EPDS	16	7.9 (4.7)	13	13.8 (7.5)	−5.9 (−10.3 to −1.5) p= 0.01	16	7.1 (4.8)	11	13.7 (6.2)	−6.8 (−11.3 to −2.2) p= 0.005
PHQ-9	16	6.2 (4.2)	13	11.8 (7.8)	−5.6 (−9.9 to −1.3) p= 0.01	16	6.2 (4.3)	11	10.7 (5.8)	−4.7 (−8.8 to −0.6) p= 0.03
Physical component of SF12 score^*^	16	34.5 (7.8)	13	38.5 (5.8)	−4.4 (−9.5 to 0.7) p= 0.09	15	39.8 (6.9)	11	44.9 (12.1)	−7.5 (−15.3 to 0.3) p= 0.06
Mental component of SF12 score^*^	16	52.1 (6.4)	13	42.9 (8.9)	10.2 (4.4 to 16.0) p= 0.001	15	49.2 (4.6)	11	42.8 (11.0)	7.5 (0.9 to 14.0) p= 0.03
Metacognitive awareness questionnaire^**^	16	44.7 (6.2)	12	41.5 (8.1)	5.1 (−0.3 to 10.5) p= 0.06	15	42 (5.8)	10	39.7 (9.7)	5.0 (−0.5 to 10.4) p= 0.07
EQ-5D*	16	0.78 (0.16)	13	0.72 (0.17)	0.06 (−0.05 to 0.18) p= 0.26	15	0.90 (0.13)	11	0.78 (0.11)	0.13 (0.03 to 0.22) p= 0.01
Prenatal Attachment Inventory	16	60.4 (3.0)	10	47.2 (3.3)	2.6 (2.69 to 22.38) p= 0.01	-	-	-	-	-

(^1^) adjusted for CIS-R score at baseline, age (< or ≥ 18) depression severity (mild, moderate or severe), current symptom duration (< or ≥ 3 months) and history of depression (yes, no).

^*^High scores reflect better quality of life.

^**^ Higher scores reflect greater cognitive awareness.

### Continuous CIS-R outcome at 33 weeks – proposed secondary

Similar results were seen at 33 weeks post-randomisation. The majority, 81.2% of the women did not have depression in the intervention group compared to 36.4% in usual care only arm (Table 2). The continuous CIS-R mean score was also lower for the intervention group compared with the usual care group at 33 weeks post-randomisation. Again, after adjustment for baseline CIS-R score and the 4 key design variables the intervention group had a mean CIS-R score that was 12.6 points lower than those in the usual care group (Table 4).

### Other outcomes at 15 and 33 weeks – proposed secondary

The results for the other secondary outcomes at 15 weeks and 33 weeks are given in Table 4. Those women who were randomised to the intervention were less depressed (on both the PHQ-9 and EPDS) compared to those who were randomised to continue with usual care. Mental health related quality of life assessed using the SF-12 mental health component score showed those in the intervention group reported better mental health at 15 and 33 weeks. Physical functioning (SF-12 physical component) improved over time but there was only weak evidence of a difference between the two groups. The EQ-5D was also administered at both follow-up time points. Both groups had similar mean scores at 15 weeks but those in the intervention group had a higher mean score at 33 weeks than those receiving only usual care indicating some evidence of better health. Mean scores on the meta-cognitive awareness questionnaire improved over time but did not differ between the groups. The prenatal attachment inventory used at 15 weeks reported women in the intervention group had a higher mean score (60.4) compared to the women receiving only usual care (47.2) indicating some evidence of an increased attachment to their baby, although these differences could have been apparent at baseline.

### Health economic data

Of the 29 women who completed their 15 weeks follow-up assessment, 24 completed a Resource Use Questionnaire (RUQ). At 33 weeks, of the 27 women who were followed up, 25 completed the RUQ, only 21 women completed RUQs at both follow-up time points. Table 5 shows the mean level of resource use, using all available data for the 33 women.

**Table 5:** Resource-use data using all available data for the 33 women

	**Maternity notes**	**GP records**	**Self report questionnaire (RUQ)**
Mean (st dev) number of GP visits per month	N/A	0.67 (0.47) *n*=33	0.45 (0.35) *n*=20
Mean (st dev) number of midwife visits per month	0.96 (0.41) *n*=29	0.46 (0.40) *n*=33	0.95 (0.60) *n*=13

In order to compare the resource-use measurement collection methods, analyses were conducted only on those women who had complete information from the comparative sources. For midwife appointments, RUQ and maternity note collection methods were comparable (Table 6). GP record extractions differed significantly from both RUQ and maternity note extractions and in terms of GP visits, GP records recorded a higher median number of GP visits than the RUQ.

**Table 6:** Comparisons of resource-use measurement collection methods (Wilcoxon signed-ranks tests)

**Per month**	***n***	**Maternity notes**	**GP records**	**Self-report RUQ**	***p*-value**
Median number GP visits per month (GP records and self report RUQ)	20	N/A	0.53	0.36	0.1
Median number midwife visits per month (GP records and RUQ)	13	N/A	0.13	0.92	0.009
Median number midwife visits per month (maternity notes and RUQ)	11	0.92	N/A	1.03	0.5
Median number midwife visits per month (GP records and maternity notes)	29	0.92	0.53	N/A	<0.001

Information from medical records and maternity notes were recorded. Medical record searches were conducted on 33 women, as 2 women withdrew from the study and 1 woman had a miscarriage. Of those 33 women, information from 29 maternity notes were collected, with the team unable to access 4 women’s maternity notes due to moving out of the area. The average number of antenatal appointments was 13 (range 7–25, SD= 4.1), with a mean of 9 midwife appointments (range= 2–21, SD= 3.6) and a mean of 3 scans (range= 1–6, SD=1.2).

### Antidepressant use

Any interventions the women received as part of usual care (in both groups) were recorded. Medical records showed that over a third (39.4%; 13/33) of the women had been prescribed antidepressants in the year before randomisation. The majority of those women (69.2%; 9/13) were taking an antidepressant during their pregnancy (2 women in the intervention, 7 usual care), however 3 women stopped taking them after giving birth (all in usual care). Of the 4 women who were not prescribed antidepressants during pregnancy, 3 women were prescribed them after giving birth (1 in the intervention, 2 in usual care).

### Referral information for the treatment of depression from medical notes

According to medical records, very few women in the trial had any referrals in relation to a diagnosis of depression in the year prior to randomisation. The majority (72.7%; 24/33) had nothing recorded in their medical notes regarding any referrals, 21.2% (7/33) had a referral made for them and only 6.1% (2/33) women had ‘discussed’ a referral with their doctor. During the study period, fewer women who received the intervention (14.3%; 1/7) had a referral compared to those in usual care (85.7%; 6/7). All those referrals were to a psychological treatment service, 5 women were referred to or assessed by a specialist perinatal psychiatry service and 2 were referred to another service, although there was no information available on whether treatment was started or completed (See Table 7).

**Table 7:** Referral information for the treatment of depression from medical notes

	**Total sample**	**Intervention**	**Usual care**
**Year prior to randomisation**	**n= 33**	**n=18**	**n=15**
Nothing recorded	24	14	10
Referral made to:	7	2	2
Psychological treatment service		1	-
Drug and Alcohol service		-	1
Crisis team A&E psychiatric team		-	1
Discussed low mood	2	1	1
**During the study period**	**n= 7**	**n= 1**	**n= 6**
Assessed on a specialist perinatal unit	3	-	3
Referred to a specialist perinatal unit	2	1	1
Referred to a psychological treatment service	1	-	1
Received phone counselling sessions	1	-	1

## Discussion

To our knowledge, this is the first trial of CBT looking at treating depression by the end of pregnancy, with the aim of assessing the feasibility of delivering a CBT intervention modified for antenatal depression during the antenatal period. The results of this pilot study suggest that it is feasible to complete CBT with some adaptation for context, before the end of pregnancy. Although the sample of the study was small, the results suggest that CBT could be effective in reducing the symptoms of antenatal depression by the end of pregnancy.

### Feasibility of recruitment

We found it was possible to recruit women through midwife booking appointments, however, midwives only referred 65.6% of those eligible for assessment. There was a significant loss from those attending booking appointments and those randomised mainly due to busy midwives not inviting women to take part in the study. Booking appointments involve the collection of a great deal of information and any future trial in this area would need to identify other additional ways of recruiting women. Posters in waiting rooms or mail outs from surgeries could be other possibilities. Midwives subsequently reported that they often did not ask women about the trial, therefore ongoing seminars and education to encourage them to refer to mental health research studies is important, as extending the reach to many of those women who were not asked to take part is essential.

### Delivery of therapy

An important aim of this study was to assess whether we could deliver CBT in a sufficiently timely fashion to show benefit before the end of pregnancy. It was therefore important that women were identified early in pregnancy and assessed rapidly. The time between booking appointments and randomisation was on average 3 weeks, although we believe this could be shortened by focusing research team resources at this point.

Based on clinical improvements in weekly self-reported depression scores collected as part of CBT sessions, the therapists judged that 8 to 9 sessions were often sufficient, however as 12 sessions were offered initially this set up an expectation that made it difficult to end earlier (for both client and therapist). We propose that for any future study that a more reasonable approach would be to ‘treat until well’ with a limit of 12 sessions, as residual symptoms may be linked to negative child outcomes. Also it is clearly important to avoid unnecessary sessions in order to maximise potential for cost effectiveness.

One aspect of this intervention which is different from standard CBT is that it was offered to women in their own homes. All but one woman took up this option which was designed to increase the accessibility of the therapy in order to maximise attendance at sessions. DNA rates for cancelled sessions were low (5.6%) and this is comparable to previous studies [47,48]. Therapy at home is particularly relevant for women who are continuing to work and those who had problems arranging childcare. Previous studies have found that women have reported that some of the major barriers to treatment are the practical ones such as lack of time, childcare issues, and being seen in a hospital setting [49,50]. However, the therapists reported that therapy seemed less formal when conducted in the home and that the setting influenced the relationship between therapist and participant in subtle ways which might influence the effectiveness of the intervention through changes in nonspecific factors (i.e. therapist as authority) of any of the intervention [51]. Therapists offered to conduct sessions even when young children were present if childcare could not be set up, however women generally did not want to do this as they felt their child would interfere with the CBT. These issues could have implications for the cost-effectiveness, as providing CBT at home reduces the capacity of the therapist. However, there is growing evidence from other studies that telephone based and internet CBT is more accessible, as acceptable and effective as face to face CBT [52-54], therefore the mode of delivery needs to be considered to ensure generalisabilty without compromising effectiveness.

### Other methodological issues

The early identification and recruitment of women (between 8 and 18 weeks) with depression during pregnancy means there will be a number of drop outs due to miscarriage before assessment, and these women are also at risk of having depression. Also those women with an onset of depression later in pregnancy will be missed. The only way to address this would be to extend the intervention to those at high risk of developing depression during pregnancy but the cost effectiveness of such an approach including an element of depression, would need to be considered.

Although this is a pilot trial and the sample was small, one potential source of bias that this pilot has highlighted is the higher dropout rate in the usual care only group. Other depression trials have reported being able to avoid differential attrition by having sufficient funds to increase contact between appointments [55]. Keeping in touch by letter or telephone between assessments might help to minimise this problem in a large trial, although such contact should be minimal (and to both groups) to avoid contamination from any potential therapeutic benefit that might arise.

### Use of resources

The numbers of women returning information on resource use was not sufficiently large to draw any confident conclusions. However, collection of data on resource use indicates that for midwife contacts self-reported resource use appears to be as reliable as maternity notes, whereas it appears that many maternity appointments are not recorded in GP records, which should be discounted as a reliable means of obtaining maternity information. In terms of GP visits, self-report appeared to under estimate contacts with GP compared with GP records; this confirms earlier work, albeit in a different patient group, that found elderly patients underestimate resource use even within a short time frame [56]. In a future trial if it is too expensive to collect information from GP records, using resource-use logs to aid memory could be considered.

## Conclusion

This pilot RCT indicates that it would be feasible to conduct a full scale trial to assess the effectiveness of CBT given in addition to usual care, compare to usual care alone, for the treatment for antenatal depression. Strategies are needed to improve referral of potentially eligible women by midwives to such a trial. The promising results from this pilot trial suggest that there could be potential benefits of such an intervention to the mother and therefore to wider family and the developing child. A sufficiently large trial to provide evidence of the effectiveness and cost effectiveness of such an intervention is needed, as this cannot be assumed by simply extrapolating from evidence outside the antenatal period.

## Abbreviations

RCT: Randomised Controlled Trial; CBT: Cognitive behavioural therapy; GP: General Practitioner; NICE: National Institute for Clinical Excellence; CIS-R: Clinical Interview Schedule – Revised version; EPDS: Edinburgh Postnatal Depression Scale; PHQ-9: The Patient Health Questionnaire; RUQ: Resource-use questionnaires.

## Competing interests

The Authors declare that they have no competing interests.

## Authors’ contributions

JE, HO, NW, PR, DS, KT were responsible for the conception and design, and securing funding for the trial. JE as principal investigator had overall management responsibility of the study. AB and HB, as trial coordinators, were responsible for the day to day running of the trial and data collection. KB was involved in the data collection. HO provided clinical supervision throughout the trial. AB led the analysis and interpretation of the data with supervision from JE and NW, drafted the article and revised the article. JT jointly designed the RUQ and analysed the health economic data under the supervision of SN. SN jointly designed the RUQ. All authors revised, read and approved the final version of the paper for publication.

## Pre-publication history

The pre-publication history for this paper can be accessed here:

http://www.biomedcentral.com/1471-244X/13/33/prepub
